# Should we be Afraid of Immune Check Point Inhibitors in Cancer Patients with Pre-Existing Rheumatic Diseases? Immunotherapy in Pre-Existing Rheumatic Diseases

**DOI:** 10.31138/mjr.32.3.218

**Published:** 2021-09-30

**Authors:** Kalliopi Klavdianou, Konstantinos Melissaropoulos, Alexandra Filippopoulou, Dimitrios Daoussis

**Affiliations:** 1 Department of Rheumatology, “Asklepieion” General Hospital, Athens, Greece; 2 Department of Rheumatology, Agios Andreas Hospital, Patras, Greece; 3 Clinical Immunology-Rheumatology Unit, 2nd Department of Medicine and Laboratory, Hippokration General Hospital, National and Kapodistrian University of Athens, Athens, Greece; 4 Department of Rheumatology, Patras University Hospital, University of Patras Medical School, Patras, Greece

**Keywords:** immune checkpoint inhibitors, cancer immunotherapy pre-existing rheumatic diseases, rheumatic diseases

## Abstract

**Background:**

Cancer immunotherapy is rapidly expanding but its clinical efficacy is hampered by immune related adverse events (ir-AE). There is a concern regarding patients with pre-existing auto-immune diseases (PAD) undergoing immunotherapy.

**Methods:**

An electronic search was performed (Medline) to identify cases of patients with PAD treated with immune checkpoint inhibitors (ICI).

**Results:**

Published data are rather limited but continue to emerge. Patients with PAD exhibit a high risk of PAD flare and/or de novo ir-AE. In most cases PAD flares and de novo irAEs were not severe and could be managed effectively with standard treatment.

**Conclusions:**

This risk in patients with PAD appears acceptable, and therefore, these patients could receive immunotherapy under close monitoring. Collaboration of oncologists and rheumatologists for the management of these patients is crucial.

## INTRODUCTION

The immune system is responsible not only for the defence against pathogens, but also for the elimination of cancer cells. The main immune cells responsible to identify and destroy cells that have undergone malignant transformation are cytotoxic T cells.^1^ The activation of T cells is a tightly controlled process. Several molecules known as immune checkpoints such as cytotoxic T lymphocyte antigen 4 (CTLA4), programmed death 1 (PD-1), and its ligand PD-L1, “restrain” T cells so that excessive or long term activation of T cells is avoided.^2^ During the last years oncologists have targeted immune checkpoints with a novel class of agents called immune checkpoint inhibitors (ICIs). These drugs act by boosting T cell activation in an effort to enhance anti-tumour immune responses and have shown a noteworthy clinical efficacy in a wide range of cancer types of advanced stage and have favoured survival outcomes in many patients worldwide.^3^ However, T-cell induced activation by ICIs leads to immune-related adverse events (irAEs).^4^ Auto-inflammatory and autoimmune toxicities have been extensively studied and may affect any organ, including the musculoskeletal system.^5–10^ Taking into account the risk of adverse events, patients with pre-existing rheumatic disease (PAD) have been excluded from the initial clinical trials of ICIs. However, later on, patients with PAD were eventually treated with ICI in everyday clinical practice. Questions regarding ICI efficacy, risk of irAEs or autoimmune disease flare in patients with PAD on immunotherapy are being raised. There is a growing amount of studies assessing the safety and efficacy of these agents in patients with malignancy and PAD. Outcomes and management of irAEs in patients with PAD are included in the recently published EULAR task force ‘points to consider’.^11^ In this review, we aimed to explore the current evidence on the safety of immunotherapy in patients with PAD.

## METHODS

An electronic search in PubMed was made until end of August 2020 by using the following key words: immune checkpoint inhibitors, cancer immunotherapy combined with pre-existing rheumatic diseases, rheumatic diseases, rheumatoid arthritis, arthritis, myositis, polymyalgia rheumatica, musculoskeletal, rheumatic, sicca, Sjogren syndrome, vasculitis, sarcoidosis, spondyloarthropathies, psoriatic arthritis, systemic lupus erythematosus, and systemic sclerosis, in various combinations. We assessed the abstracts of the articles and identified those describing patients with cancer and pre-existing rheumatic diseases treated with immunotherapy. Reports regarding patients with autoimmune but not rheumatic diseases such as multiple sclerosis and autoimmune thyroiditis were excluded. We included only studies in English language and published as full articles without setting time limits. Outcomes of immunotherapy in patients with PAD are categorised according to disease type.

## RHEUMATOID ARTHRITIS (RA)

RA is the most frequent rheumatic disease and usually affects the small joints of the hands.^12^ T cells appear to play a role in the pathogenesis of the disease since down-regulating T cell activation by blocking co-stimulation is an effective therapeutic approach in RA. Abatacept is an approved therapy for RA; it is a fusion protein consisted of CTLA4 attached to the Fc region of IgG1 and acts by blocking co-stimulation signals and therefore inhibiting activation of T cells.^13^ On the other hand, in Oncology, CTLA4 is also targeted but in a different way; the monoclonal antibody ipilimumab blocks CTLA4 and leads to enhanced T cell activation.^1,2^ Taking the above into account, there is certainly a theoretical concern regarding the use of immunotherapy, especially in the form of anti-CTLA4, in patients with RA. This is why patients with RA were initially excluded from ICI clinical trials.^14–16^ As a result, limited and mainly retrospective data have thus far been provided.

Tison et al. reported 20 patients with previously diagnosed RA. At the onset of therapy with ICI agents, 8 patients had high disease activity. Mainly mild flares were observed in 60% (12/20) of patients, while irAEs were reported in 35% (7/20). Five (5/20) subjects had a combination of flare and irAEs. Therapeutically, a low dose of steroids was administrated, while ICIs discontinuation was required only in 15% (3/20).^17^

Menzies et al. reported 13 patients with RA who underwent treatment with ICIs. Among them, 5 patients had active disease, when treatment with anti-PD1 was initiated. Mild flares, mainly involving joint pain were more commonly reported. Symptoms were controlled with low dose steroids, occasionally demanding administration of steroid sparing agents, while treatment with ICIs was continued.^15^

Leonardi et al. included 11 patients with a prior history of RA in their case studies. Active disease was observed in 27% (3/11) of them at the time of ICI initiation. A mild flare, requiring symptomatic therapy, was observed in 55% (6/11). ICIs were not discontinued. Only one patient exhibited a grade 3 exacerbation of arthritis, concurrent with nephritis; steroids were administrated, while immunotherapy was temporarily interrupted. Two more patients with pre-existing RA exhibited colitis and thyroiditis, successfully controlled with steroids and levothyroxine, respectively.^18^

Lee et al. reported 8 patients with pre-existing RA who received treatment with ipilimumab. An exacerbation of arthritis was reported in 6 of these patients. More specifically, a grade 1 flare was observed in four subjects and was managed by administration of non-steroidal anti-inflammatory drugs (NSAIDs) in standard doses. Two subjects exhibited a grade 3 flare, requiring high doses of steroids with rapid tapering. Of note, 4 subjects were hospitalized because of colitis indicating that patients with prior history of RA may present a higher rate of irAEs.^19^

Johnson et al. reported 6 patients with previously diagnosed RA who underwent treatment with ipilimumab. Five of these 6 patients exhibited joint pain, most frequently 2 to 3 weeks following immunotherapy initiation. Two of them had parallelly developed hypophysitis and colitis, respectively. The patient, who did not report joint pain, eventually developed thyroiditis. Arthralgia was successfully controlled with low dose of steroids and immunotherapy was not discontinued.^14^

Richter et al. reported 5 patients with prior history of RA. In most patients (4/5), immunotherapy was well tolerated, without toxicities, whereas a single patient developed colitis.^20^

Danlos et al reported two subjects with a prior history of RA which both experienced an irAE; however, more details were not provided.^21^ Finally, Gutzmer et al. reported a single patient with a previous diagnosis of RA who developed a mild flare of arthritis (without concurrent irAEs) 2 weeks following ICI administration. Steroids were effective, with no need for ICI discontinuation.^22^

Eleven more subjects with pre-existing RA were reported in 4 cohort studies, even though complete data were not always available. Exacerbation was often observed, clinically improved by low doses of corticosteroids for at least 6 months.^23^ Despite the frequent and occasionally severe irAEs ^24,25^ discontinuation of ICI agents was avoided in most cases.^23–26^

According to available data, out of 66 patients with pre-existing RA treated with ICI a flare of arthritis was observed in 37/66 (56%), usually mild. IrAEs were developed at a lower rate (30%). Furthermore, flares were concurrent with irAEs in 8/66 (12%) patients.

Based on the above we may conclude that patients with RA exhibit a higher risk for both de novo ir-AE and PAD flare. However, this risk does not appear unacceptably high and therefore exclusion of RA patients from immunotherapy does not appear justified.^14–16^ Patients with RA with low disease activity and not requiring high doses of immunosuppressants may carry a lower risk.^15,16,22^ Anti-PD1 agents are more highly recommended for patients with RA since they are considered to exhibit a lower grade of toxicity compared to anti-CTLA4.^15^ Close monitoring of these patients is of importance for early diagnosis and treatment of potential irAEs. In general, flares of RA under immunotherapy can be managed effectively with low/moderate dose of steroids without discontinuation of ICIs.^14,15,22^

## POLYMYALGIA RHEUMATICA

Polymyalgia rheumatica (PMR) is a common inflammatory rheumatic disease affecting the elderly.^27^ PMR has been often described as a musculoskeletal irAE following ICI administration.^6^ There is a limited amount of data concerning patients with previously diagnosed PMR undergoing immunotherapy.

Johnson et al. reported a flare of PMR, rapidly and successfully treated with high dose of prednisolone (30mg/day, tapered over one month.^14^

Danlos et al. described a case of one patient with PMR treated with methotrexate as steroid sparing agent. He was subsequently diagnosed with metastatic melanoma, therefore DMARD administration was discontinued and nivolumab was initiated. De novo irAEs were not observed, however cancer progression was lethal.^21^

An exacerbation of pre-existing PMR, diagnosed 15 months before melanoma, was also reported in the case series by Gutzmer et al. The patient was under treatment with steroids when PMR relapsed, 5 weeks following anti-PD-1 infusion. Prednisolone was administrated in order to control the flare.^22^

Menzies et al. report 3 patients who experienced anti-PD-1–induced flare of PMR. Generally, most cases did not require discontinuation of ICI therapy and disease exacerbations were mainly controlled by administration of oral steroids.^15^

Richter et al. reported 5 patients with pre-existing PMR. Two of them experienced irAEs. More precisely, one patient with coexistent giant cell arteritis (GCA) presented with a flare of temporal arteritis, after 1 cycle of nivolumab infusion. The second patient exhibited ipilimumab-induced colitis. Both of them required steroids.^20^

Pre-existing PMR was reported in 5 patients by Leonardi et al. Mild flares were reported in 60% (3/5) of subjects, not requiring specific treatment. Steroids were administrated in a single case of more severe muscle pain, accompanied by arthralgia. None of these 5 patients exhibited irAE and ICIs were not discontinued.^18^

Seven patients with prior history of PMR/GCA were included in the case series by Tison et al. Active disease was present in 29% (2/7) of them, at ICI initiation. A mild PMR flare was observed in 43% (3/7) of patients, all requiring steroids. Furthermore, 4 patients also experienced irAEs, resulting in temporary ICI discontinuation in 29% of cases.^17^

Mitchell et al. reported 7 patients with previously diagnosed PMR, following therapy with ICIs. Four of them (57%) had an IrAE, demanding steroids for at least 6 months. Disease flares were observed in 86% (6/7) of subjects, appearing at a median time of 6 weeks following ICI initiation.^23^

Overall, 30 patients with previously diagnosed PMR underwent therapy with ICIs. Exacerbation was exhibited in 17/30 (57%) of the reported subjects, whereas irAEs were observed in 33% of them. Generally, flares of underlying PMR are more often observed than de novo irAEs, usually requiring low dose steroids,^17,23^ with no need for ICI discontinuation.^15^

## PSORIATIC ARTHRITIS

Most studies addressing the issue of ICI therapy in patients with PAD have included cases of psoriasis and the distinction between cases of psoriasis and cases of psoriatic arthritis (PsA) is not always evident. Tison et al. studied a mixed cohort of 31 cases of psoriasis with or without PsA and found that 21/31 patients experienced a flare, manifesting mainly as recurrence of psoriatic plaques, and 13/31 had a de novo irAE.^17^ Active disease at baseline was recorded in 17/31; of these patients 60% (10/17) experiencing a flare. Immunosuppression with prednisone<15mg/day, methotrexate or acitretin was required in 7 cases.

Regarding patients with a relatively clear diagnosis of PsA the following data are presented. In the study by Johnson et al, 2 patients with PsA were reported. One patient was on methotrexate (MTX) and did not experience a flare during follow up.^14^ The other patient had an exacerbation from the skin and also developed severe colitis, for which treatment with high-dose steroids was implemented. In the study by Menzies et al., one out of two patients with PsA experienced a flare.^15^ Both patients had active disease at baseline. Mitchell et al.,^23^ reported two patients with pre-existing PsA in their case-series. Both of them had a grade 2 flare, manifesting as peripheral joint pain. Treatment with steroids was sufficient and one patient also received MTX which had a steroid-sparing effect. Both patients continued anti-PD1 therapy with at least partial cancer response. An interesting case of PsA was described in the study by Calabrese et al., with a patient previously treated with apremilast.^28^ The disease was quiescent while on chemotherapy, but an exacerbation of psoriasis happened soon after nivolumab initiation. Apremilast was re-administered and anti-PD1 treatment was continued with mild psoriasis lesions and without joint inflammation during follow-up. In the study by Kähler et al.,^29^ one patient with psoriasis and arthritis, receiving low dose prednisolone at inclusion, had a skin exacerbation without the need for baseline treatment intensification. Leonardi et al. reported two cases of PsA treated with anti-PD1 therapy for lung cancer, with 1/2 experiencing a flare of arthritis which was managed with prednisolone, oxycodone and NSAIDs.^18^ This patient did not have to stop immunotherapy, in contrast to the second case who had a serious grade 3 immune mediated hepatitis and was treated with systemic steroids. In another cohort, one patient had an exacerbation of pre-existing PsA, despite baseline MTX treatment.^30^ A single-centre cohort reported one patient with PsA who experienced a flare, without the need for immunotherapy discontinuation.^26^ Danlos et al. reported one case of psoriasis who developed psoriatic arthritis and pustular psoriasis after immunotherapy initiation.^21^ In a case report, one patient with a history of psoriatic arthritis on MTX had a severe skin exacerbation following MTX discontinuation and pembrolizumab initiation.^31^ The patient did not have arthritis and responded to acitretin and phototherapy regarding skin disease, managing to restart immunotherapy.

To sum up, 11/14 (78%) patients with psoriatic arthritis experienced some type of flare of their PAD. Existing evidence indicate a high PAD flare rate in psoriasis/PsA that could be linked to the pathogenetic role of T cells in psoriasis and PsA.^32^ However, it is important to note that these patients mainly experienced exacerbation from the skin and severe articular manifestations rarely occurred. Standard treatment with topical therapy, steroids and DMARDs was often sufficient. Permanent termination of immunotherapy was not necessary in most cases.

## MYOSITIS

Patients with known inflammatory myopathy starting ICI therapy are extremely rare. In the study by Gutzmer et al., a 54-year-old male patient with advanced melanoma, carrying a myositis diagnosis without receiving treatment at baseline, experienced a grade 3 flare 16 weeks after starting pembrolizumab.^22^ The patient was treated with intravenous immunoglobulin, apparently with good results as anti-PD1 therapy was continued. Tison et al., reported a patient with dermatomyositis, being active at baseline and receiving prednisone at a dose of 5mg/day when started nivolumab treatment, who did not experience a flare during follow-up.^17^ In a case report, a 73-year-old woman with a history of autoimmune myositis and Crohn’s disease in remission received pembrolizumab for lung cancer.^33^ No PAD flare was reported but the patient developed a grade 4 irAE, manifesting as severe neutropenia.

The data regarding ICI therapy in a background of pre-existing inflammatory myopathy are too scarce to provide recommendations. Each case should be individualized and caution is warranted. We should also note that myositis in cancer patients might be a paraneoplastic manifestation and not an idiopathic rheumatic disease.

## SPONDYLOARTHRITIS

Spondyloarthritis (SpA) refers to a group of inflammatory diseases with a clinical spectrum consisting of spine and peripheral joint/tendon symptoms, as well as extra-articular manifestations. Patients reported to have a diagnosis belonging to this spectrum appear in some cohorts. Tison et al. reported five cases of patients with SpA, who were treated with anti-PD1 therapy.^17^ One patient who had stopped anti-TNF treatment 4 months before ICI initiation had active disease at baseline. A second patient was on low dose steroids when ICI was started. During follow-up, 2/5 cases experienced a flare with axial symptoms. Among them was the case who had stopped anti-TNF treatment. This patient was afterwards treated with high dose steroids and NSAIDs. Another patient developed a de novo serious irAE and had to stop immunotherapy. Two studies concerning melanoma cohorts had a possible overlap in their cases.^22,29^ One cohort was treated with anti-CTLA4 and the other with anti-PD1 inhibitors. We identified four patients with SpA and only 1/4 experienced a grade 2 flare treated with steroids and NSAIDs. One patient with concurrent psoriasis and ankylosing spondylitis was receiving MTX, etanercept and prednisolone at baseline. This patient did not experience a flare or irAE. Another three case-series reported altogether four patients with a history of SpA receiving ICI.^20,21,30^ Exacerbation of the underlying rheumatic disease was not recorded.

Collectively, 3/13 (23%) patients with a SpA diagnosis had a flare after ICI initiation and 1/13 developed a definite serious irAE. Even though several factors including baseline treatment could be important, patients with SpA seem to have a low risk of severe exacerbation after ICI initiation perhaps owing to the autoinflammatory rather than autoimmune background of this group of diseases.

## VASCULITIS

Data regarding patients with pre-existing vasculitis on ICI come from a few case reports and retrospective studies based on registries. No flare of pre-existing ANCA-associated vasculitis in 2 patients was reported in a retrospective study.^17^ In the same cohort 3 out of 7 patients with PMR/GCA on steroids experienced a PAD flare managed with steroids with no need for DMARDs. Three patients experienced irAE while 1 patient experienced both flare and irAE. One patient with pre-existing polyarteritis nodosa from the Registry of Severe Adverse Reactions to Immunomodulatory Antibodies in Oncology (REISAMIC) in France did not develop an irAE after anti-PD1 treatment.^21^ In a retrospective study, one patient with GCA presented with nephritis after ICI initiation.^18^ The AE was resolved with steroids and ICI discontinuation. A flare of cranial symptoms in an 89-year-old female with GCA after treatment with nivolumab was reported in a database of Mayo clinic.^34^ In the same study a flare of granulomatosis with polyangiitis (GPA) in a 68-year-old male with melanoma on ipilimumab was identified. GPA flare was managed with cyclophosphamide and intravenous steroids. Four patients with vasculitis were identified in a large health insurer database of ICI-treated patients in the US. PAD was associated with a modest increase in hospitalizations with irAE and with steroid treatment. Flare of pre-existing disease was not included in study outcomes.^35^

No flare of eosinophilic granulomatosis with polyangiitis (EGPA) and GPA after anti-PD1 treatment was described in two case reports.^36,37^ Nabel et al. described the case of a 56-year-old male with GPA with a biopsy proven pulmonary granuloma, after anti-PD1 initiation for urothelial carcinoma. The patient received steroids and rituximab for the GPA flare.^38^ Due to the rarity of this entity, no safe conclusions can be drawn regarding irAEs and risk of PAD flare in patients with vasculitis. We may hypothesize that PAD remission prior to ICI treatment could yield a lower risk of flares.

## SARCOIDOSIS

There are limited published data regarding patients with pre-existing sarcoidosis receiving ICIs. In a multicentre study, 2 patients with sarcoidosis starting anti-PD1 were not initially on treatment for the autoimmune disease. One patient experienced flare of sarcoidosis and the other immune related pneumonitis, both treated with steroids.^22^ In another retrospective study, one out of two patients with sarcoidosis and melanoma on anti-CTLA4 experienced disease exacerbation with hypercalcemia.^14^ A case of sarcoidosis deterioration with hypercalcemia treated with steroids in a patient with skin cancer on anti-PDL1 has also been reported.^39^ In a study by Sakakida et al., 2 patients with pre-existing untreated sarcoidosis experienced no worsening.^40^ One out of two patients developed thyroid dysfunction after anti-PD1 treatment. Together, 19% and 25% of patients reported in literature experienced a relapse of the pre-existing autoimmune disease or de novo irAEs, respectively.^14,17,20–22,26,30,39,40^ Data on patients on ICIs with pre-existing sarcoidosis indicate that PAD flare or irAEs could happen but can be managed with steroids.

## SICCA-SJÖGREN’S

Reports regarding possible ir-AEs or disease flare in patients with pre-existing Sjögren’s Syndrome (SS) are scarce. In a series of 52 patients with PAD on anti PD1-therapy, both patients with SS included, had an active disease at baseline and experienced a flare.^15^ In a retrospective study by Mayo Clinic, 2 patients with SS and inactive disease at treatment initiation were identified. One out of 2 developed interstitial pneumonitis after anti-PD1, managed with steroids and cancer treatment switching.^20^ In the REISAMIC registry 1 out of 4 patients with pre-existing SS developed a disease flare.^21^ All four patients with SS on anti-PD(L)1 identified in other retrospective studies did not experience PAD flare or de novo irAE.^17,18^ Overall, a PAD flare occurred in one fourth of patients with SS on ICIs and an irAE in only one out of 8 patients identified in literature.^15,17,18,20,21^ indicating that patients with SS may tolerate immunotherapy well.

## SYSTEMIC LUPUS ERYTHEMATOSUS (SLE)

In a multicentre study from the USA regarding patients on ICIs and PAD, one out of four patients with SLE experienced a flare. The patient was managed with steroids and temporary ICI discontinuation.^30^ Only one out of four patients with SLE identified in a retrospective study had a mild flare.^17^ In a retrospective study on patients with cancer and PAD who received a PD-(L)1 inhibitor one patient with SLE was identified.^18^ The patient experienced cutaneous and joint flare after ICI initiation, managed with topical corticosteroids, prednisone, and temporary anti-PDL-1 discontinuation. In two retrospective studies none of the four patients with SLE experienced flare or de novo irAE following ICI initiation.^14,20^ Sporadic cases of patients with SLE on ICIs have been published describing either no flare,^41^ or disease flare with neuropsychiatric symptoms similar to neuropsychiatric lupus.^42^

In several cohorts, nine cases of cutaneous lupus have been identified.^17,18,21,26,30^ One flare and three de novo irAEs have been reported. Data regarding patients with SLE on immunotherapy are limited and solid conclusions cannot be drawn. Based on the existing evidence the risk for flare or de novo ir-AE appears acceptable and therefore the exclusion of SLE patients from immunotherapy is not justified.

## SYSTEMIC SCLEROSIS (SSC)

In most retrospective cohorts, no flare of SSc was reported.^17,18,23^ Flare of pre-existing SSc after anti-PD1 has been reported in a melanoma patient.^15^ In a retrospective multicentre study of patients with metastatic melanoma on ipilimumab and PAD, a patient with limited SSc experienced both a disease flare and colitis,^29^ and was treated with steroids. Given the rarity of the cases of patients on ICIs with pre-existing SSc, it is difficult to make conclusions regarding possible flares.

## PATIENTS WITH POSITIVE ANA

During the last years there has been some concern regarding whether patients with positive autoantibodies are more prone to develop irAE following treatment with ICI and whether they have better or worse cancer outcomes. So far data are rather limited and conflicting. ANAs have been associated with an improved outcome in patients with colorectal cancer and lung cancer on ICIs.^43,44^ A retrospective study evaluating 137 patients with lung cancer who received ICI also showed that patients with autoantibodies had higher progression free survival compared to patients with no autoantibodies.^45^ However, the presence of these autoantibodies was correlated with irAE development. On the other hand, a recent study showed different results regarding progression free survival.^46^ The authors analysed 83 patients, of whom 18 were ANA positive, that received ICI. The incidence of irAEs did not differ significantly between ANA negative versus positive patients. Progression-free survival and overall survival were significantly shorter in ANA positive patients compared to ANA negative patients in this study. Positive ANA have been found in 9 out of 191 patients with various cancers receiving ICIs, in a retrospective study by Sakakida et al.^40^ ANA positivity prior to ICI treatment, did not predict the development of irAEs with the exception of colitis. None of the 9 patients with ANA ≥1:160 before ICI initiation, developed SS, SLE, or myositis. Three of the 4 patients who developed irAE after ICI therapy, were ANA positive before treatment.^47^ Based on the above inconclusive results, EULAR states that there is no indication to test every patient for autoantibodies before ICI treatment.^11^

Rates for PAD flares and de novo irAEs in patients with PAD are presented in **Table 1**.

**Table 1.** Cases of pre-existing autoimmune diseases (PAD) on immune-checkpoint inhibitor (ICI) therapy retrieved from literature.

**PAD**	**Cases, n**	**Flare, n (%)**	**De novo irAE, n (%)**	**Refs**

Rheumatoid arthritis	66	37 (56)	20 (30)	[14–15], [17–22]

Polymyalgia rheumatica	30	17 (57)	10 (33)	[14–15], [17–18], [20–23]

Psoriatic arthritis	14	11 (79)	3 (21)	[14–15], [18], [21], [23], [26], [28–31]

Inflammatory myopathy	3	1 (33)	1 (33)	[17], [22], [33]

Spondyloarthritis	13	3 (23)	1 (8)	[17], [20–22], [29–30]

Vasculitis				
*ANCA-associated*	6	2 (33)	2 (33)	[17–18],
*Polyarteritis nodosa*	1	0 (0)	0 (0)	[21],
*Giant cell arteritis*	6	4 (67)	5 (83)	[34–38]

Sarcoidosis	16	3 (19)	4 (25)	[14], [17], [20–22], [26], [30], [39–40]

Sicca-Sjögren’s	12	3 (25)	1 (8)	[15], [17–18], [20–21]

Systemic lupus erythematosus	15	4 (27)	0 (0)	[14], [17–18], [20], [30], [41–42]

Systemic sclerosis	9	1 (11)	0 (0)	[15], [17–18],[23], [29]

## DISCUSSION

The use of immunotherapy is rapidly expanding. Patients with rheumatic diseases constitute a significant percentage of the general population and therefore oncologists frequently face the challenge of deciding whether cancer patients with PAD are suitable for immunotherapy. The existing evidence clearly indicate that patients with PAD carry an increased risk for both PAD flare and de novo irAEs. In most cases, PAD flares and de novo irAEs were not severe and could be managed effectively with standard treatment. Taking into account that immunotherapy may be extremely effective in specific patients, oncologists should not exclude patients from treatment with ICI solely based on a PAD diagnosis. From a practical point of view, oncologists should obtain a detailed history and record any PAD prior to initiation of immunotherapy. Detection of autoantibodies does not provide any meaningful information and is not recommended. In case oncologists are uncertain about the nature or the severity of the PAD, then an expert opinion of a rheumatologist could be obtained. This is of importance especially in patients with a recent PAD diagnosis and/or receiving immunomodulating therapy. In these cases, rheumatologists should assess the severity and activity of PAD; in patients receiving immunosuppressive therapy they should adjust treatment in a way that the patient is treated with the lowest immunosuppressive regimen possible to maintain PAD under control. If treatment with steroids is needed, they should remain at a dosage of less than 10mg prednisone/day to maintain ICI efficacy.^11^ In cases where patients with PAD are not receiving any kind of immunosuppressive therapy at the initiation of immunotherapy, then, only close monitoring is needed. In general, agents targeting PD-1/PD-L1 axis associate with less irAE compared to anti-CTLA4 therapy,^4^ and could be preferred for the treatment of patients with PAD. On the other hand, the combination therapy with both types of ICI is associated with significantly more irAEs and should be avoided, if possible, in these patients.

As immunotherapy advances new immune checkpoints and novel treatment combinations are being explored. The therapeutic strategy of combining different classes of ICI leads to a robust activation of the immune system and associates with enhanced clinical efficacy. However, this approach is hampered by the frequency and severity of irAEs which limit the clinical use of several powerful combinations of immunotherapy agents. Recently, a novel therapeutic approach is explored in animal models aiming at minimizing irAE triggered by combination immunotherapy. The researchers used a mouse model and showed that TNF inhibition before the start of anti-CTLA-4 and anti-PD-1 combination therapy prevents autoimmune adverse events, and partially enhances the anti-tumour efficacy of the combined treatment.^48^ Mice injected with cancer cell lines intraperitoneally and treated with anti-CTLA4 plus anti-PD1, developed autoimmune colitis following dual checkpoint inhibition. Prophylactic treatment with anti-TNF increased the infiltration of tumour-specific CD8 T cells in mice tumours and ameliorated autoimmune colitis. These results provide a strategy dissociating ICI efficacy and toxicity and may have promising translational implications. The use of pre-emptive immunosuppressive treatment could be a way of minimising irAEs, especially in high risk patients such as patients with severe PAD or active PAD at baseline. The current prevailing point of view is that immunosuppression diminishes the efficacy of immunotherapy. However, the experimental evidence presented above challenges this view and indicates that immunosuppressants such as TNF blockers are not only effective in controlling severe irAEs but may also associate with enhanced oncologic response. Up until data from humans are available pre-emptive immunosuppression cannot be recommended; patients should be treated only when irAE appear and with the lowest dose needed to control manifestations.

Up until now we do not have reliable biomarkers to predict which patients under immunotherapy will develop irAEs, even though some progress has been made in this area.^49^ These biomarkers could be particularly useful in the management of high-risk patients such as those with PAD and could pinpoint patients with exceedingly high risk of irAE.

In conclusion, patients with PAD exhibit a high risk of PAD flare and/or de novo irAE. This risk appears acceptable and therefore these patients could receive immunotherapy under close monitoring. Collaboration of oncologists and rheumatologists for the management of these patients is crucial.

## ABBREVIATIONS

ANA: Antinuclear Antibodies
CTLA4: Cytotoxic T Lymphocyte Antigen 4
DMARD: Disease-modifying anti-rheumatic drug
EGPA: Eosinophilic Granulomatosis with Polyangiitis
ICI: Immune Check point Inhibitor
Ir-AE: Immune related Adverse Event
GCA: Giant cell arteritis
GPA: Granulomatosis with polyangiitis
NSAID: Nonsteroidal Anti-inflammatory Drug
PAD: Pre-existing autoimmune diseases
PD-1: Programmed Death 1
PD-L1: Programmed Death Ligand
PMR: Polymyalgia Rheumatica
PsA: Psoriatic Arthritis
RA: Rheumatoid Arthritis
SLE: Systemic Lupus Erythematosus
SpA: Spondyloarthritis
SS: Sjogren’s syndrome
SSc: Systemic Sclerosis
TNF: Tumour Necrosis Factor
